# Patients and Nurses: Complaints of Familiarity

**Published:** 1916-09-16

**Authors:** 


### Patients and Nurses: Complaints of Familiarity.

Sir,—I was pleased to read in The Hospital of September 9 your remarks on the attitude adopted by some nurses towards their patients in addressing them by such familiar terms (however kindly intended) as "Granny," "Daddy," etc., and I have heard many patients speak (though not in every case actually complaining) in terms of comparison and expressing great appreciation of being addressed by their proper names.

My personal feeling is that our hospitals are the cradles of the trained nurse, and if she is allowed there to acquire this unfortunate mode of address she will find it difficult to forget it when she comes to deal with patients in their own homes. My work lies entirely amongst the poor, and I could cite many instances where the patients have shown their dislike of this familiarity. In one case quite recently the man who was so sensitive about it, instead of telling the nurse "not to call again," sent for his clergyman to ask him to tell nurse not to continue calling him "Daddy." In another case the patient, an unmarried man, well educated, but in very reduced circumstances through no fault of his own, criticised this habit in quite a severe manner.

But I would plead for one step further, and that is the cessation of the objectionable and universal custom of calling every woman in the humbler walks of life by the term "Mother." I went with a young unmarried woman who had come up from the country to consult about her sister-in-law's child, and she was more than surprised to be continually addressed as "Mother."

I do hope your paper will draw the attention of those who are responsible for guiding our younger nurses into the straight path, to the great desirability of giving due deference to the poor as well as to the rich by a proper and respectful mode of address.—I am, yours, etc., Custom.

